# Supplementary material for: Dimerization of the S. cerevisiae Spo11 core complex
Source: bioRxiv. 2026 Jan 17:2026.01.16.699991. Preprint. [Version 1] doi: 10.64898/2026.01.16.699991 (PMC12871325; doi:10.64898/2026.01.16.699991)
Supplement: 1 [file NIHPP2026.01.16.699991V1-supplement-1.pdf]

bioRxiv preprint doi: https://doi.org/10.64898/2026.01.16.699991; this version posted January 17, 2026. The copyright holder for this preprint (which was not certified by peer review) is the author/funder, who has granted bioRxiv a license to display the preprint in perpetuity. It is made available under aCC-BY 4.0 International license.

Supplementary Files

Dimerization of the *S. cerevisiae* Spo11 core complex

Hajar Aït Bella[1], Mahesh Survi[1], Julian Urdiain-Arraiza[1], Dayishaa Daga[2], Vijayalakshmi V. Subramanian[2], Andreas Hochwagen[2] and Corentin Claeys Bouuaert[1,*]

[1] Louvain Institute of Biomolecular Science and Technology, Université catholique de Louvain, 1348 Louvain-La-Neuve, Belgium.
[2] Department of Biology, New York University, New York, NY 10003, USA

* Correspondence to corentin.claeys@uclouvain.be

Supplementary Figures

Supplementary Figure 1: Quality assessment of AlphaFold models of dimeric Spo11 core complexes with and without DNA.
Supplementary Figure 2: Comparison of the AlphaFold3 model and the cryo-EM structure of *S. cerevisiae* Spo11 core complex.
Supplementary Figure 3: Structure-informed alignment and conservation of Spo11 orthologs.
Supplementary Figure 4: Structure-informed alignment and conservation of Ski8 orthologs.
Supplementary Figure 5: Structure-informed alignment and conservation of Rec102 orthologs.
Supplementary Figure 6: Structure-informed alignment and conservation of Rec104 orthologs.
Supplementary Figure 7: DNA-binding properties of Spo11 dimerization mutants.
Supplementary Figure 8: Conservation of Spo11 and Top6A dimer interfaces.
Supplementary Figure 9: The Spo11-A236V mutant does not affect DNA binding and dimerization.
Supplementary Figure 10: Mutagenesis analysis of positively charged Rec104 residues.
Supplementary Figure 11: Impact of Rec102-K129E mutation on the DNA-binding activity of the core complex.
Supplementary Figure 12: Structural modeling of Spo11 core complexes in different fungi.

Supplementary Tables

Supplementary Table 1: Input sequences and quality scores of AlphaFold models.
Supplementary Table 2: List of plasmids.
Supplementary Table 3: List of oligonucleotides.
Supplementary Table 4: List of yeast strains.

bioRxiv preprint doi: https://doi.org/10.64898/2026.01.16.699991; this version posted January 17, 2026. The copyright holder for this preprint (which was not certified by peer review) is the author/funder, who has granted bioRxiv a license to display the preprint in perpetuity. It is made available under aCC-BY 4.0 International license.

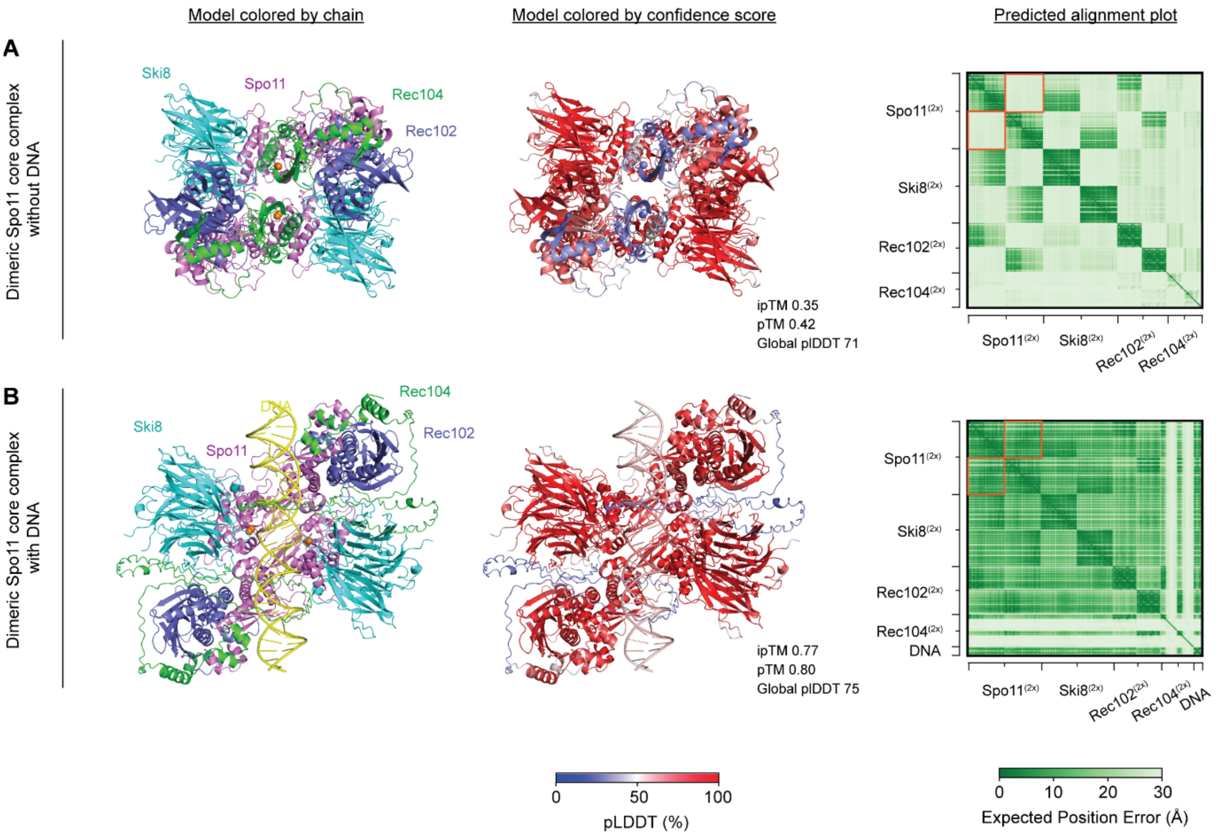

Supplementary Figure 1: Quality assessment of structural models of dimeric Spo11 core complexes with and without DNA. AlphaFold3 models of 2:2:2:2 Spo11:Ski8:Rec102:Rec104 dimeric core complexes without (**A**) and with (**B**) a 40 bp duplex DNA substrate (5'-CGCACACACATCACACACCGCGGTGTGTGATGTGTGTGCG-3'). Left: AlphaFold3 models colored by chain. Spo11 (violet), Ski8 (cyan), Rec102 (blue), Rec104 (green), DNA (yellow) and Mg^{2+} ions (orange). Middle: AlphaFold3 models colored by confidence score. Left, predicted alignment error plot. In both models, the overall structure of Rec104 is predicted with low confidence, while those of Spo11, Ski8 and Rec102 are predicted with high confidence. The confidence in the relative position of Spo11 from Complex1 with Spo11 from Complex2 is indicated by the orange squares on the predicted alignment error plots. (**A**) In the absence of DNA, the relative position of the two Spo11:Ski8:Rec102:Rec104 heterotetramers is predicted with low confidence, and AlphaFold3 predicts an aberrant Spo11 dimer interface. (**B**) In the presence of DNA, the relative position of the two copies of the core complex is predicted with high confidence, and AlphaFold3 predicts a reliable Spo11 dimer interface.

bioRxiv preprint doi: https://doi.org/10.64898/2026.01.16.699991; this version posted January 17, 2026. The copyright holder for this preprint (which was not certified by peer review) is the author/funder, who has granted bioRxiv a license to display the preprint in perpetuity. It is made available under aCC-BY 4.0 International license.

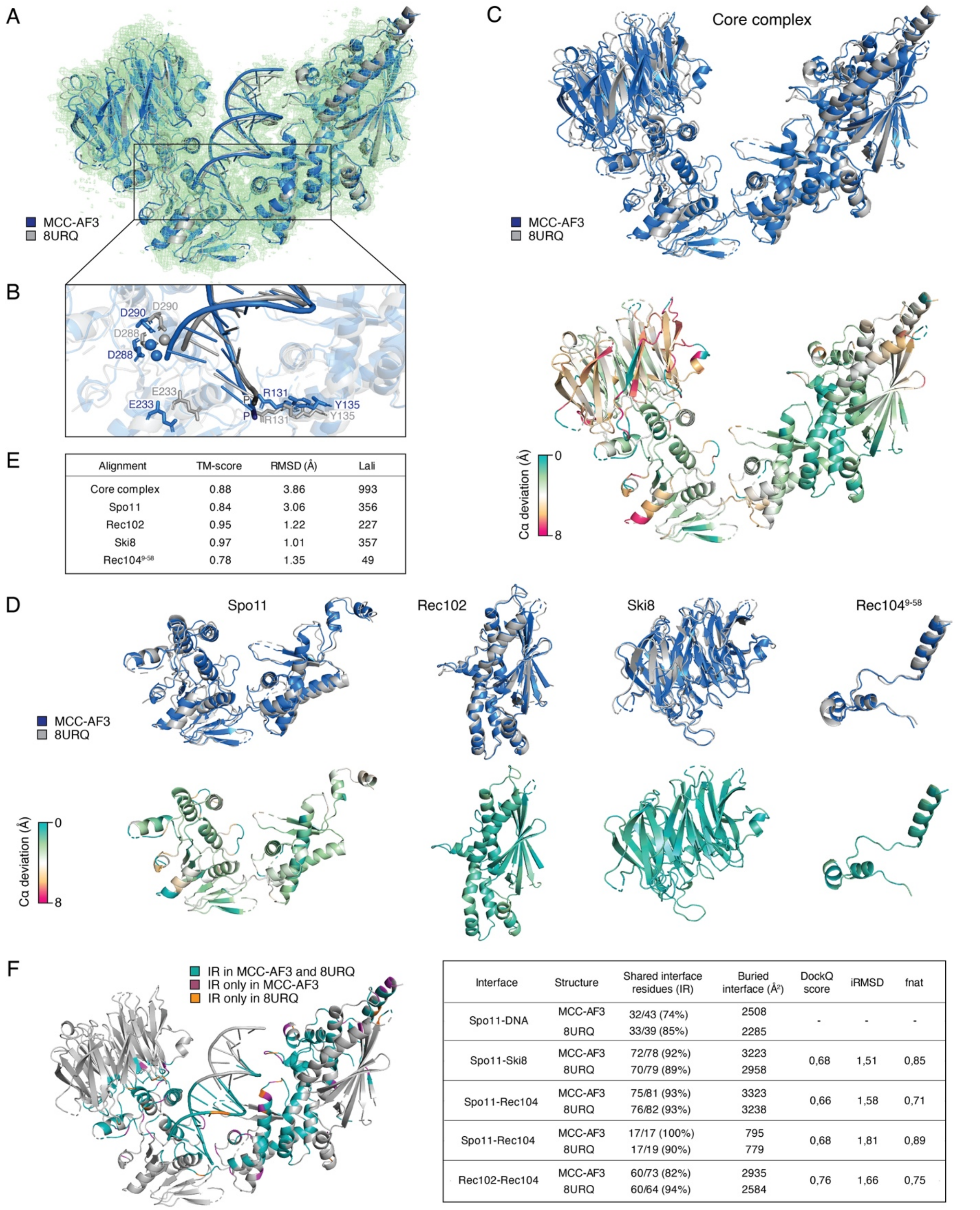

E

Alignment	TM-score	RMSD (Å)	Lali
Core complex	0.88	3.86	993
Spo11	0.84	3.06	356
Rec102	0.95	1.22	227
Ski8	0.97	1.01	357
Rec104$^{9-58}$	0.78	1.35	49

F

Interface	Structure	Shared interface residues (IR)	Buried interface (Å^2)	DockQ score	iRMSD	fnat
Spo11-DNA	MCC-AF3	32/43 (74%)	2508	-	-	-
	8URQ	33/39 (85%)	2285			
Spo11-Ski8	MCC-AF3	72/78 (92%)	3223	0,68	1,51	0,85
	8URQ	70/79 (89%)	2958			
Spo11-Rec104	MCC-AF3	75/81 (93%)	3323	0,66	1,58	0,71
	8URQ	76/82 (93%)	3238			
Spo11-Rec104	MCC-AF3	17/17 (100%)	795	0,68	1,81	0,89
	8URQ	17/19 (90%)	779			
Rec102-Rec104	MCC-AF3	60/73 (82%)	2935	0,76	1,66	0,75
	8URQ	60/64 (94%)	2584			

Supplementary Figure 2. Structural analysis and comparison of the *S. cerevisiae* monomeric core complex predicted by AlphaFold3 (MCC-AF3) and determined by cryo-EM (PDB: 8URQ).

(**A-F**) MCC-AF3 corresponds to the Complex 1 of the *S. cerevisiae* dimeric core complex predicted by Alphafold3 (DCC-AF3, **Figure 1E**) and used throughout this work. MCC-AF3 was trimmed to the resolved regions present in 8URQ. (**A**) Superposition of DNA-bound core complex of MCC-AF3 (blue) and 8URQ (gray) in the electron density map of 8URQ (green, EMDB EMD-42497 nominal 3.3 Å map). (**B**) Active site comparison of MCC-AF3 (blue) and 8URQ (gray). Mg^{2+} ions are shown as spheres. Active site residues (R131, Y135, E233, D288, D290) are shown as sticks. Scissile phosphates (P) are in dark blue (MCC-AF3) or in dark gray

bioRxiv preprint doi: https://doi.org/10.64898/2026.01.16.699991; this version posted January 17, 2026. The copyright holder for this preprint (which was not certified by peer review) is the author/funder, who has granted bioRxiv a license to display the preprint in perpetuity. It is made available under aCC-BY 4.0 International license.

(8URQ). (**C**) Top: Superposition of the core complex of MCC-AF3 (blue) and 8URQ (gray). Bottom: residue-wise Cα deviations (0–8 Å) between MCC-AF3 and 8URQ were mapped onto MCC-AF3 for clear visualization. (**D**) Top: per chain superposition of Spo11, Rec102, Ski8, Rec104 from MCC-AF3 (blue) and 8URQ (gray). Bottom: residue-wise Cα deviations (0–8 Å) between MCC-AF3 and 8URQ were mapped onto CC-AF3 for clear visualization. (**E**) Core complex and per chain (Spo11, Rec102, Ski8, Rec104) US-align analysis of MCC-AF3 and 8URQ. MCC-AF3 vs 8URQ shows strong global agreement (TM-score = 0.88) with RMSD = 3.6 Å over 993 aligned residues and per chain agreement with TM-scores 0.78-0.97 and RMSD 1.1-3.1. (**F**) Interface comparison of MCC-AF3 and 8URQ subunits. Interface preservation: MCC-AF3 shares 88% of its total interface residues with 8URQ. On average, the amount of buried interface of MCC-AF3 and 8URQ subunits is similar at 93%. Interfaces were also analyzed using DockQ v2 (Mirabello and Wallner 2024). DockQ analysis over four native interfaces (Spo11-Rec102, Spo11–Ski8, Spo11-Rec104, Rec102-Rec104) yields scores of 0.66–0.75 (overall 0.71), with iRMSD 1.5–1.8 Å, and fnat 0.70–0.89 demonstrating that MCC-AF3 recapitulates the interface geometry observed in 8URQ. TM-score, Template Modeling score; RMSD, root-mean-square deviation; Lali, alignment length; iRMSD, interface RMSD; fnat, fraction of native contacts.

bioRxiv preprint doi: https://doi.org/10.64898/2026.01.16.699991; this version posted January 17, 2026. The copyright holder for this preprint (which was not certified by peer review) is the author/funder, who has granted bioRxiv a license to display the preprint in perpetuity. It is made available under aCC-BY 4.0 International license.

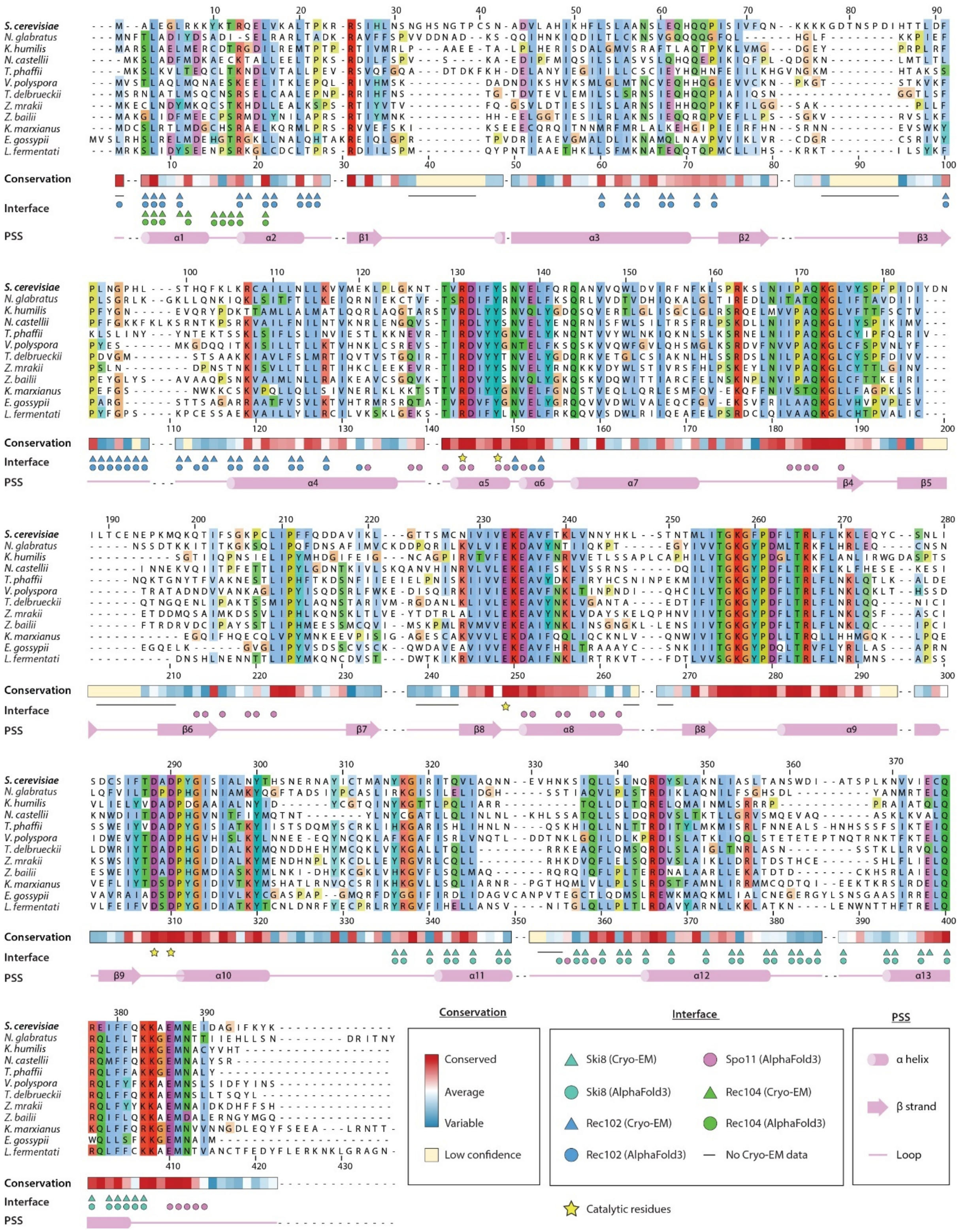

Supplementary Figure 3: Structure-informed alignment and conservation of Spo11 orthologs.
Residues of the Spo11 dimer interface (**Figure 2**) are indicated by pink circle. UniProt references: *S. cerevisiae* (P23179), *N. glabratus* (Q6FWU6), *K. humilis* (A0AAV5RW59), *N. castellii* (G0VGL2), *T. phaffii* (G8BWK5), *V. polyspora* (A7TTH3), *T. delbrueckii* (G9A007), *Z. mrakii* (A0A7H9B6N5), *Z. bailii* (A0A8J2TC47), *K. marxianus* (W0TCD2), *E. gossypii* (Q75A93), *L. fermentati* (A0A1G4M6H9). The numbering of Spo11 residues from *S. cerevisiae* is above the alignment, and the numbering of aligned residues is below the alignment. Conservation and interface data were calculated as described in Methods. PSS, protein secondary structure.

bioRxiv preprint doi: https://doi.org/10.64898/2026.01.16.699991; this version posted January 17, 2026. The copyright holder for this preprint (which was not certified by peer review) is the author/funder, who has granted bioRxiv a license to display the preprint in perpetuity. It is made available under aCC-BY 4.0 International license.

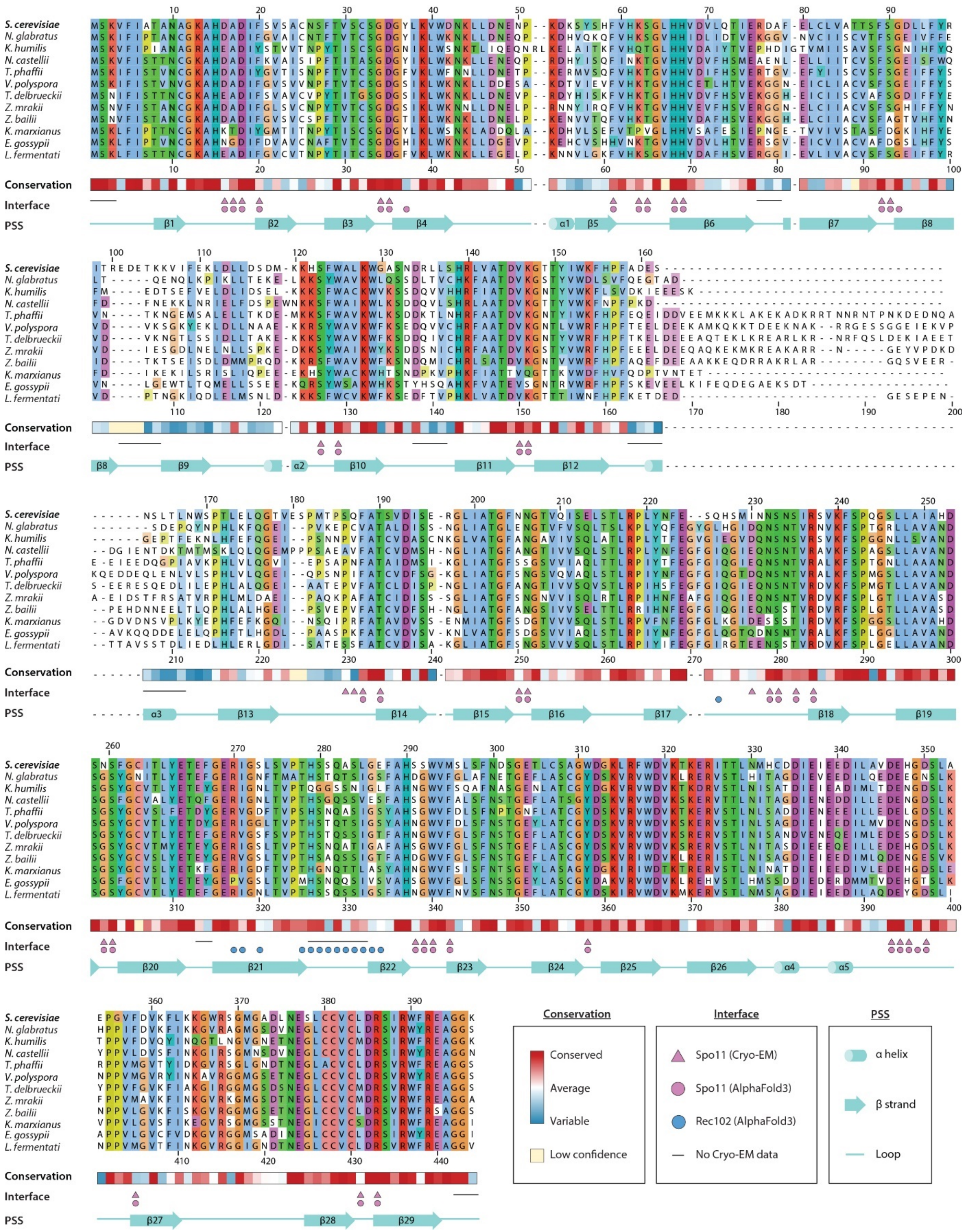

Supplementary Figure 4: Structure-informed alignment and conservation of Ski8 orthologs.
Residues of Ski8 in *trans* contact with Rec102 (**Figure 9**) are indicated by blue circles. UniProt references: *S. cerevisiae* (Q02793), *N. glabratus* (Q6FQD7), *K. humilis* (A0AAV5RRP0), *N. castellii* (G0VF58), *T. phaffii* (G8BP31), *V. polyspora* (A7TER9), *T. delbrueckii* (G8ZU84), *Z. mrakii* (A0A7H9AYH9), *Z. bailii* (A0A8J2X0D3), *K. marxianus* (W0TCZ4), *E. gossypii* (Q755S9), *L. fermentati* (A0A1G4MG77). The numbering of Ski8 residues from *S. cerevisiae* is above the alignment, and the numbering of aligned residues is below the alignment. Conservation and interface data were calculated as described in Methods. PSS, protein secondary structure.

bioRxiv preprint doi: https://doi.org/10.64898/2026.01.16.699991; this version posted January 17, 2026. The copyright holder for this preprint (which was not certified by peer review) is the author/funder, who has granted bioRxiv a license to display the preprint in perpetuity. It is made available under aCC-BY 4.0 International license.

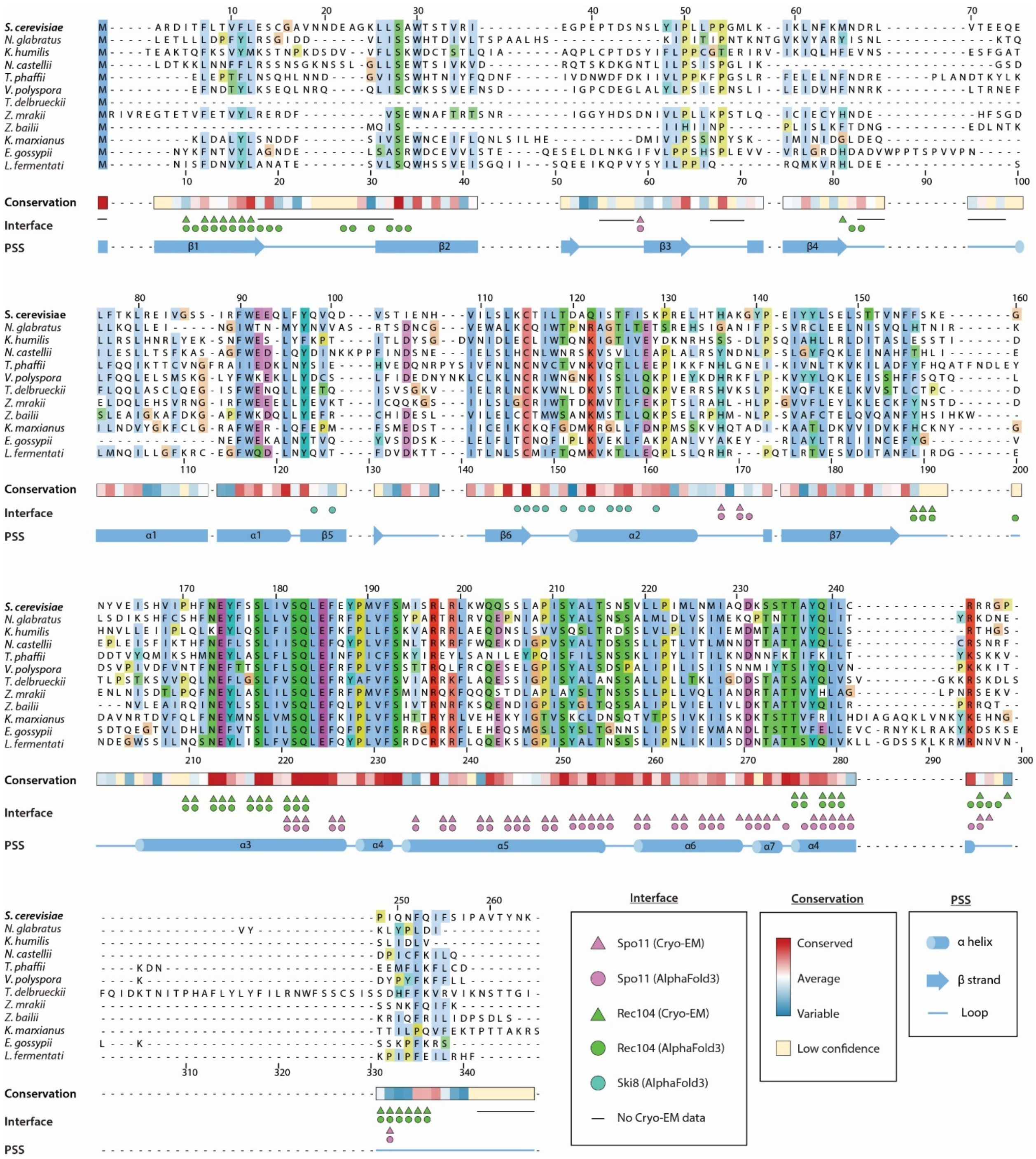

Supplementary Figure 5: Structure-informed alignment and conservation of Rec102 orthologs.
Residues of Rec102 in *trans* contact with Ski8 (**Figure 8**) are indicated by cyan circles. UniProt references (unless otherwise specified): *S. cerevisiae* (Q02721), *N. glabratus* (A0A0W0C7A9), *K. humilis* (the sequences on Uniprot A0AAV5S006 and NCBI GMM57189.1 are incorrect due to the presence of a mis-annotated intron, which was corrected here), *N. castellii* (G0VKM6), *T. phaffii* (G8BUZ4), *V. polyspora* (A7TKV1), *T. delbrueckii* (G8ZTD8), *Z. mrakii* (A0A7H9B4G4), *Z. bailii* (NCBI: AQZ11626), *K. marxianus* (W0TB81), *E. gossypii* (Q750D8), *L. fermentati* (A0A1G4MAP2). The numbering of Rec102 residues from *S. cerevisiae* is above the alignment, and the numbering of aligned residues is below the alignment. Conservation and interface data were calculated as described in Methods. PSS, protein secondary structure.

bioRxiv preprint doi: https://doi.org/10.64898/2026.01.16.699991; this version posted January 17, 2026. The copyright holder for this preprint (which was not certified by peer review) is the author/funder, who has granted bioRxiv a license to display the preprint in perpetuity. It is made available under aCC-BY 4.0 International license.

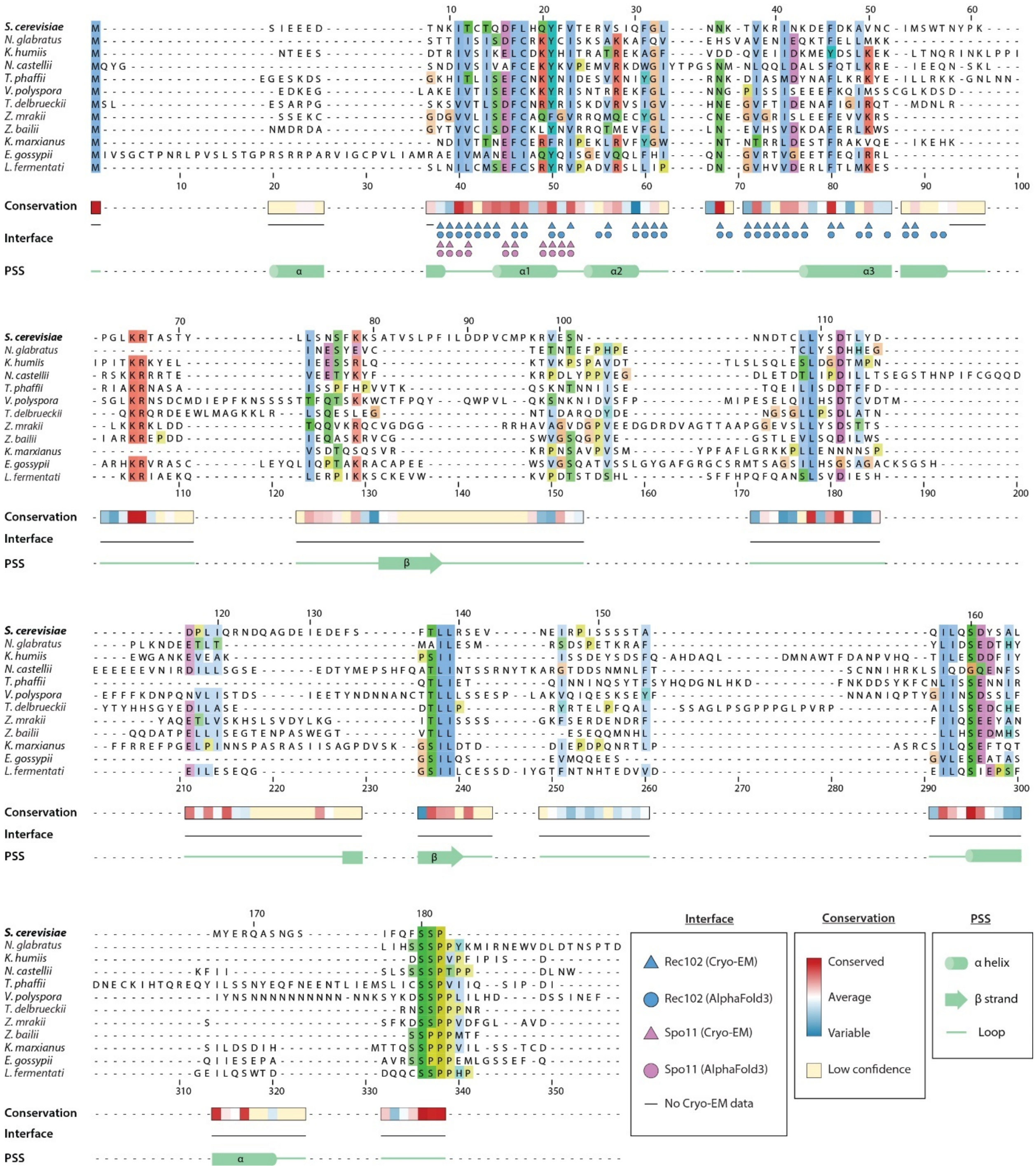

Supplementary Figure 6. Structure-informed alignment and conservation of Rec104 orthologs.
UniProt references (unless otherwise specified): *S. cerevisiae* (P33323), *N. glabratus* (Q6FR03), *K. humilis* (A0AAV5S5B3), *N. castellii* (G0VDU1), *T. phaffii* (G8BNW3), *V. polyspora* (A7TEW4), *T. delbrueckii* (G8ZYK5), *Z. mrakii* (A0A7H9B3P3), *Z. bailii* (NCBI: SJM83633.1), *K. marxianus* (NCBI: BAP72788.1), *E. gossypii* (Q75AR8), *L. fermentati* (A0A1G4MAB8). The numbering of Rec104 residues from *S. cerevisiae* is above the alignment, and the numbering of aligned residues is below the alignment. Conservation and interface data were calculated as described in Methods. PSS, protein secondary structure.

bioRxiv preprint doi: https://doi.org/10.64898/2026.01.16.699991; this version posted January 17, 2026. The copyright holder for this preprint (which was not certified by peer review) is the author/funder, who has granted bioRxiv a license to display the preprint in perpetuity. It is made available under aCC-BY 4.0 International license.

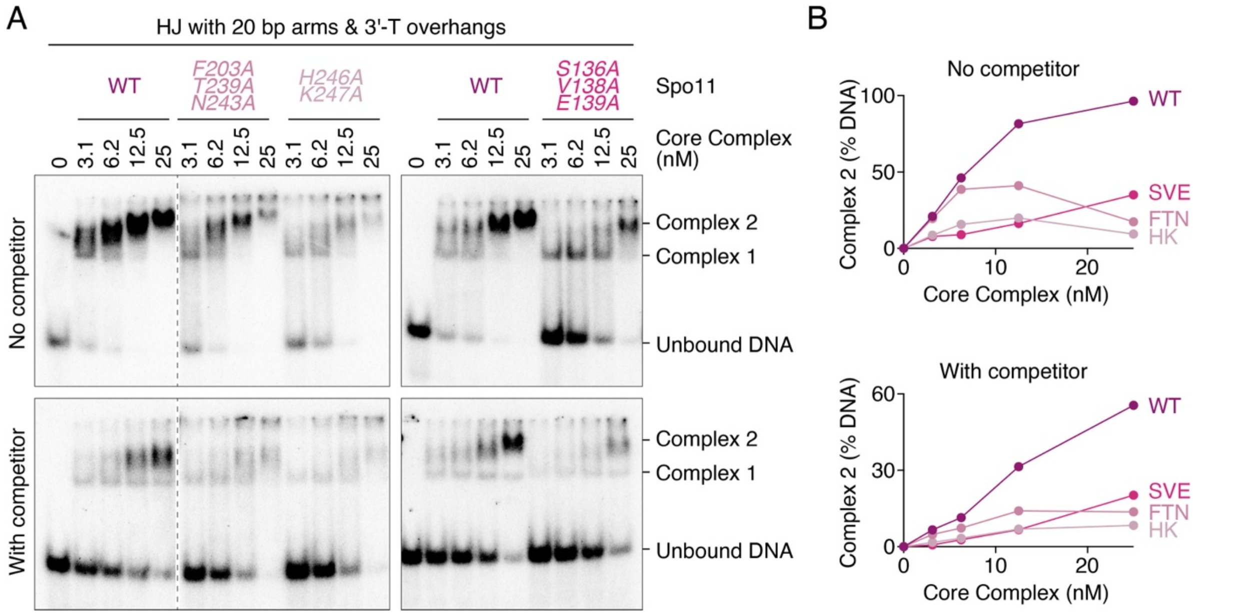

Supplementary Figure 7: DNA-binding properties of Spo11 dimerization mutants.
(A) Gel shift analysis of DNA binding by wild-type and mutant core complexes on a radiolabeled Holliday Junction (HJ) substrate (0.5 nM) with or without 20 bp dsDNA competitor (25 nM). **(B)** Quantification of Complex 2 from the gels in panel A. SVE, Spo11-S136A/V138A/E139A; FTN, Spo11-F203A/T239A/N243A; HK, Spo11-H246A/K247A.

bioRxiv preprint doi: https://doi.org/10.64898/2026.01.16.699991; this version posted January 17, 2026. The copyright holder for this preprint (which was not certified by peer review) is the author/funder, who has granted bioRxiv a license to display the preprint in perpetuity. It is made available under aCC-BY 4.0 International license.

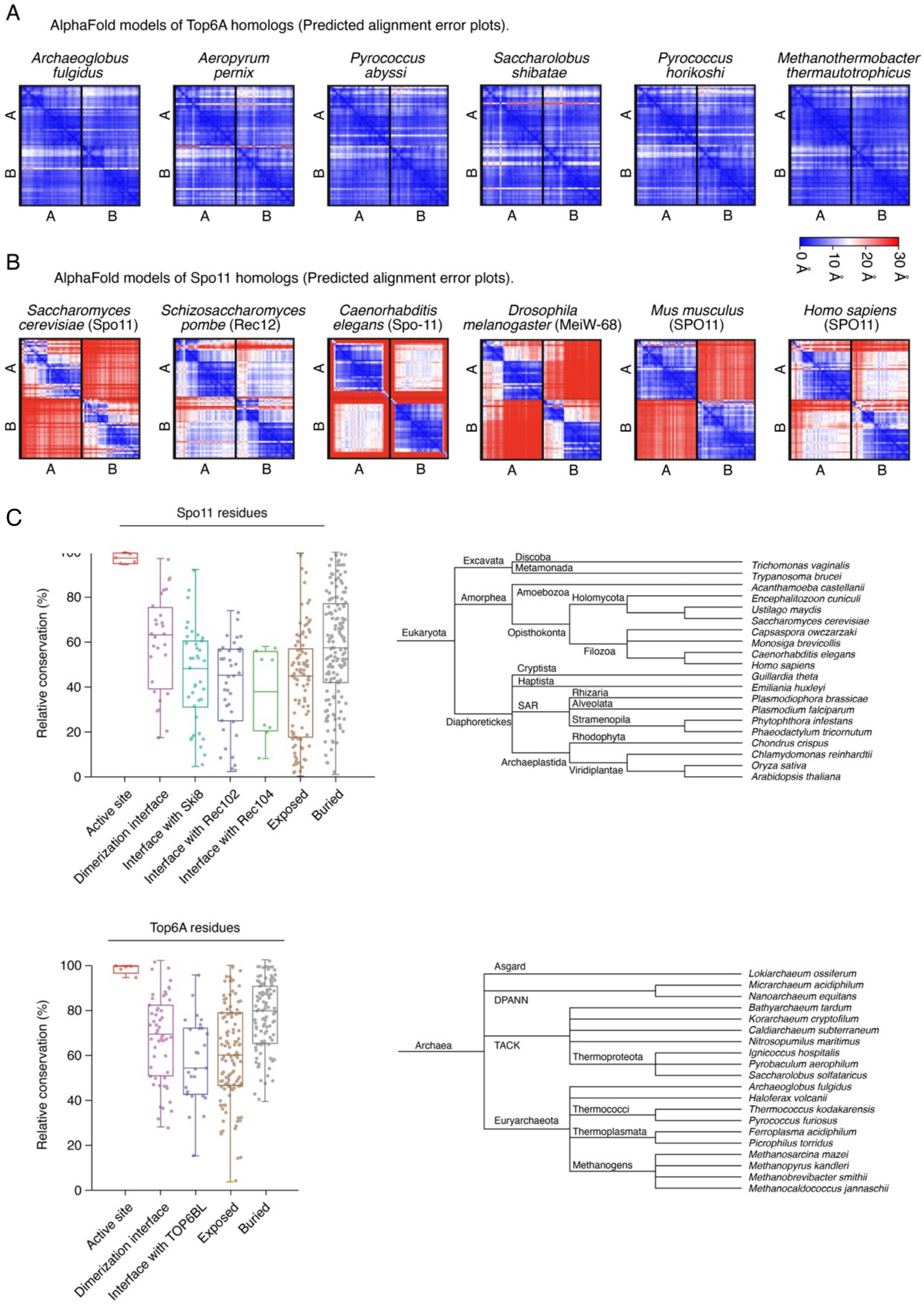

Supplementary Figure 8: Modeling of Spo11 and Top6A dimers and conservation of dimer interfaces.
(A, B) Predicted alignment error plots from AlphaFold2 models of Top6A and Spo11 dimers from selected species. The structure and relative position of two Top6A subunits are consistently predicted with high confidence. In contrast, the relative position of two Spo11 subunits (top right and bottom left squares) tend to be predicted with low confidence. **(C)** Sequence conservation analysis of Spo11 proteins across eukaryotes and Top6A proteins across archaea. (Right) Phylogenetic trees of the queried species. (Left) Sequences were aligned to *S. cerevisiae* Spo11 or *M. mazei* Top6A and residues were categorized as exposed, as buried, as parts of the active site or as parts of interaction surfaces based on the AlphaFold model of the core complex (**Figure 1E**) or a crystal structure of Topo VI (2Q2E, Corbett et al. 2007), respectively.

bioRxiv preprint doi: https://doi.org/10.64898/2026.01.16.699991; this version posted January 17, 2026. The copyright holder for this preprint (which was not certified by peer review) is the author/funder, who has granted bioRxiv a license to display the preprint in perpetuity. It is made available under aCC-BY 4.0 International license.

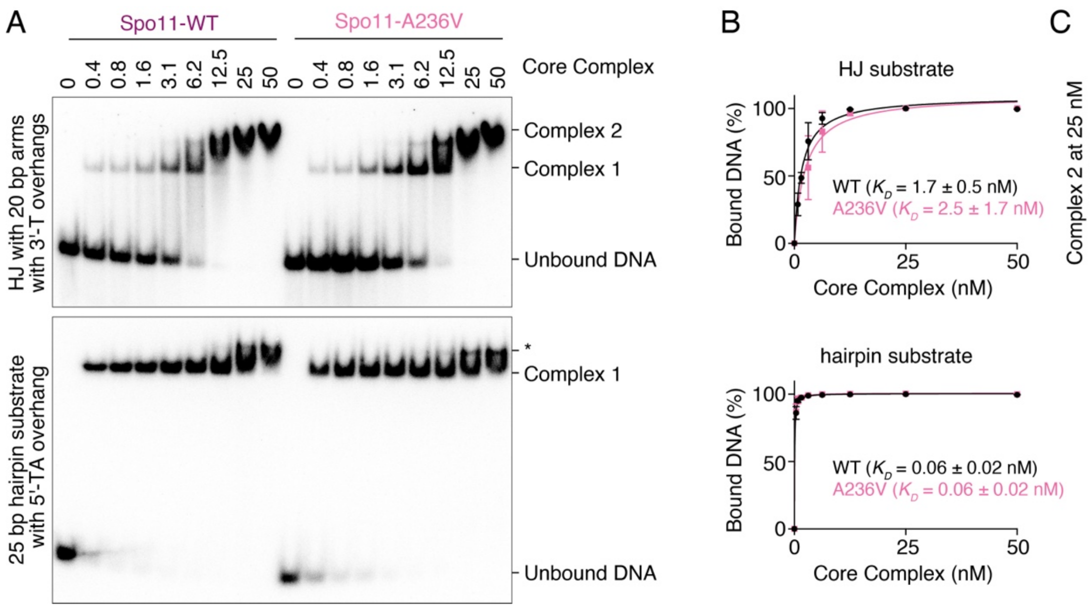

Supplementary Figure 9: The Spo11-A236V mutant does not affect DNA binding and dimerization.
(A) Gel shift analysis of the binding activity of wild-type core complexes and Spo11-A236V mutants to Holliday junctions (HJ with 20 bp arms and 1-nt 3′-overhangs), or DNA ends (25 bp hairpin substrates with a 2-nt 5′-overhang). **(B)** Quantification of the affinity of wild-type and A236V mutants for each substrate. **(C)** Quantification of Complex 2 at 25 nM core complexes (mean ± range of two independent experiments).

bioRxiv preprint doi: https://doi.org/10.64898/2026.01.16.699991; this version posted January 17, 2026. The copyright holder for this preprint (which was not certified by peer review) is the author/funder, who has granted bioRxiv a license to display the preprint in perpetuity. It is made available under aCC-BY 4.0 International license.

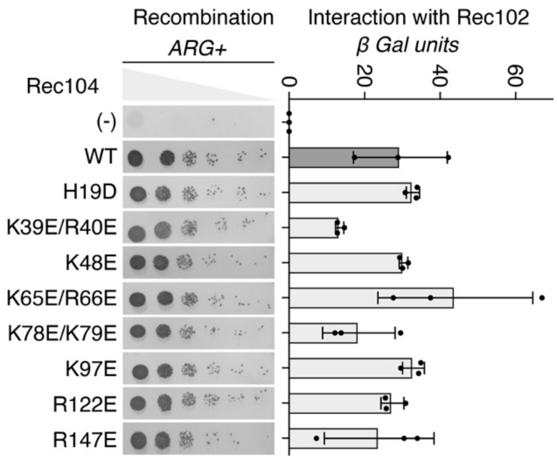

Supplementary Figure 10: Mutagenesis analysis of positively charged Rec104 residues.
Effects of Rec104 mutations on (left) recombination established by *arg4* heteroallele recombination assay and on (right) the interaction with Rec102 established by yeast two-hybrid assay (mean ± SD of three biological replicates).

bioRxiv preprint doi: https://doi.org/10.64898/2026.01.16.699991; this version posted January 17, 2026. The copyright holder for this preprint (which was not certified by peer review) is the author/funder, who has granted bioRxiv a license to display the preprint in perpetuity. It is made available under aCC-BY 4.0 International license.

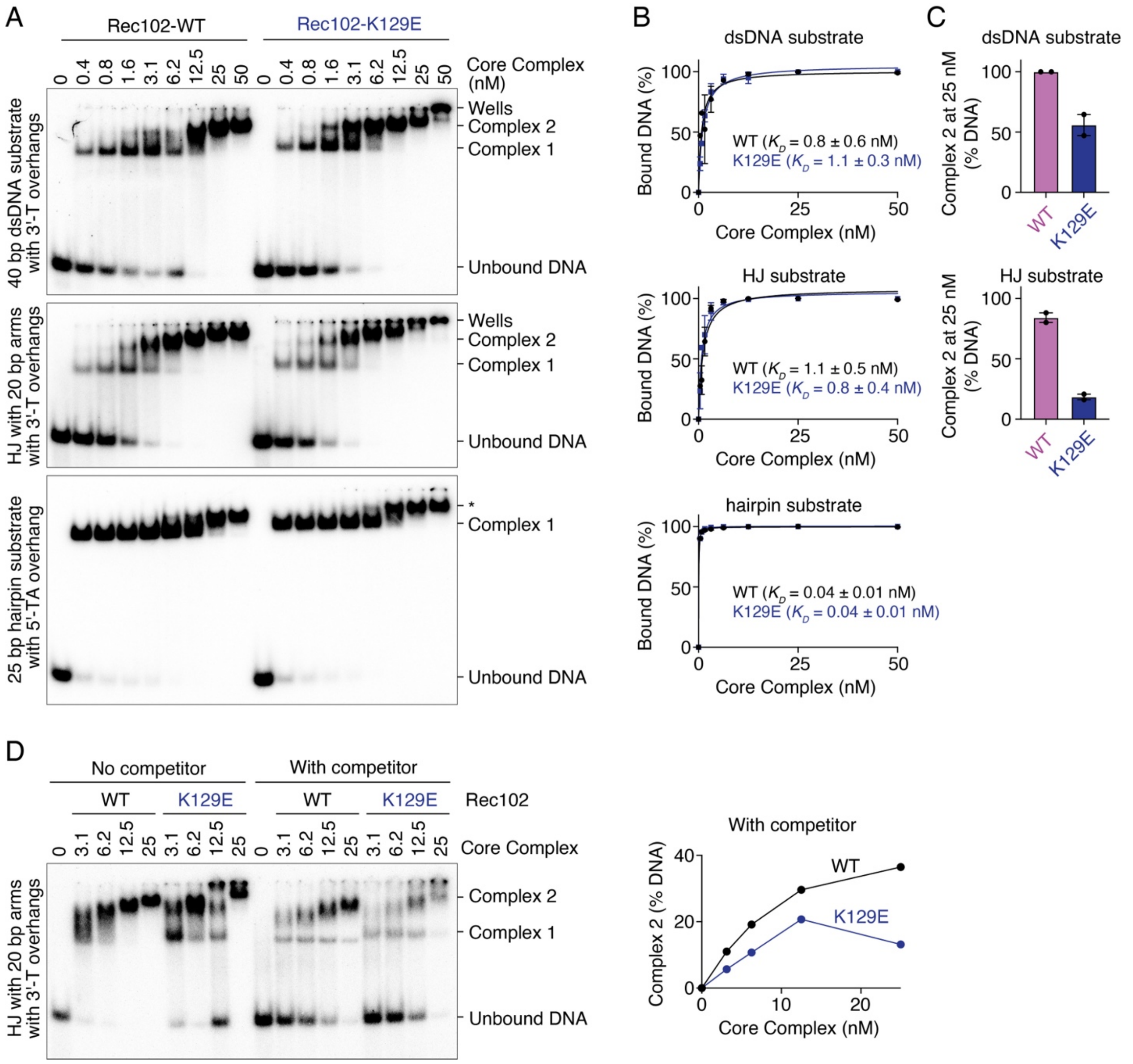

Supplementary Figure 11: Impact of Rec102-K129E mutation on the DNA-binding activity of the core complex.
(A) Gel shift analysis of the binding activity of wild-type core complexes and Rec102-K129E mutants to DNA duplexes (40 bp dsDNA with 1-nt 3′-overhangs), Holliday junctions (HJ with 20 bp arms and 1-nt 3′-overhangs), or DNA ends (25 bp hairpin substrates with a 2-nt 5′-overhang). **(B)** Quantification of the affinity of wild-type and K129E mutants for each substrate. **(C)** Quantification of Complex 2 at 25 nM core complexes (mean ± range of two independent experiments). **(D)** Gel shift analysis of binding to a labeled HJ substrate (0.5 nM) in the presence or absence of a 20 bp dsDNA competitor (25 nM). The assembly of Complex 2 in the presence of competitor is plotted.

bioRxiv preprint doi: https://doi.org/10.64898/2026.01.16.699991; this version posted January 17, 2026. The copyright holder for this preprint (which was not certified by peer review) is the author/funder, who has granted bioRxiv a license to display the preprint in perpetuity. It is made available under aCC-BY 4.0 International license.

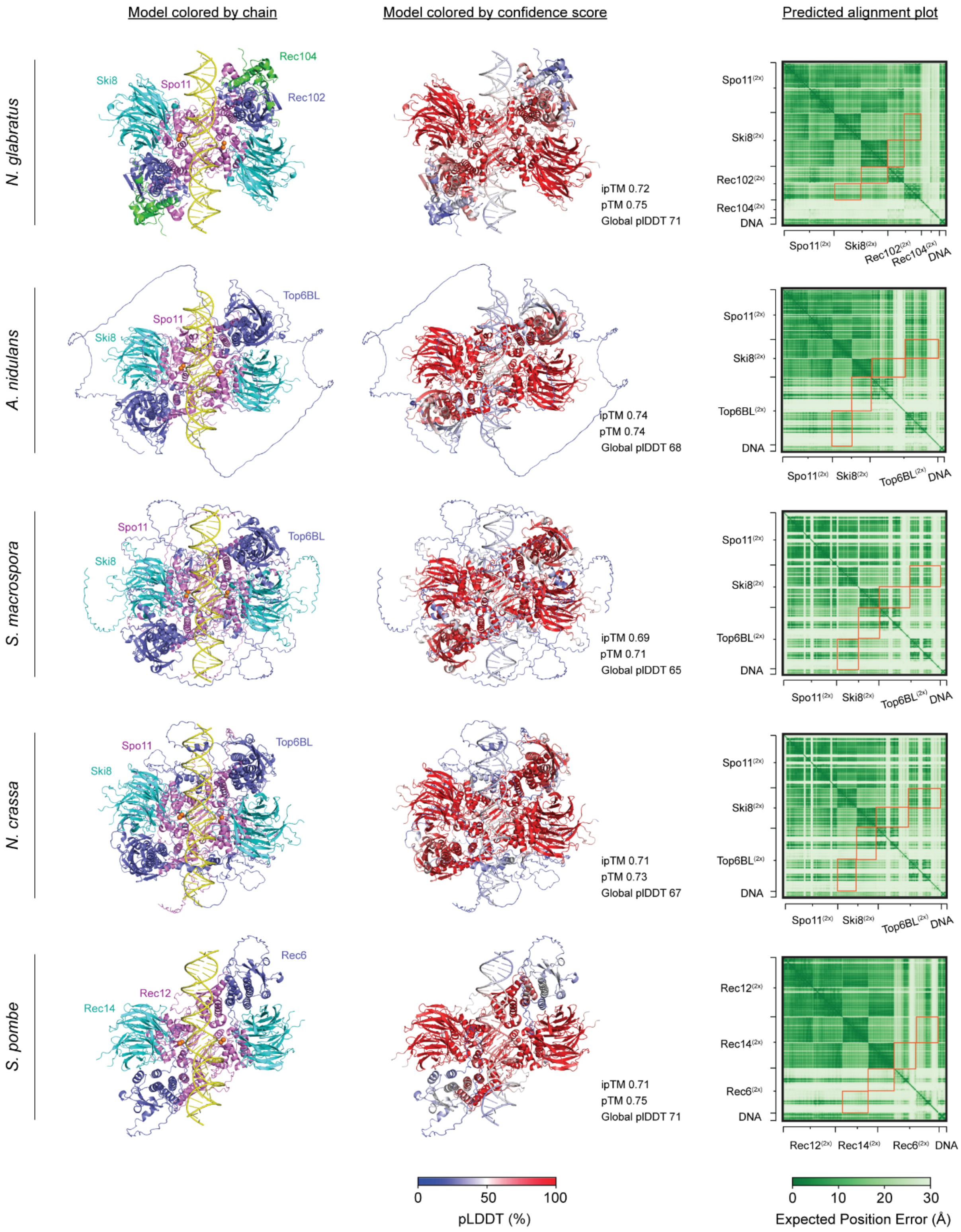

Supplementary Figure 12. Structural modeling of Spo11 core complexes in different ascomycetes.
AlphaFold3 models of DNA-bound dimeric Spo11 core complexes from *Nakaseomyces glabratus* (Q6FWU6, A0A0W0C7A9, Q6FR03, Q6FQD7), *Aspergillus nidulans* (Q5ATX1, Q5BDJ3, Q5B4V3) *Sordaria macrospora* (Q6WRU4, F7VPQ4 re-annotated, Q6URC5), *Neurospora crassa* (Q1K767, Q1K546, Q7RVN5) and *Schizosaccharomyces pombe* (P40384, P40385, Q09150). Left: AlphaFold3 models colored by chain. Spo11 homologs are shown in pink; Ski8 homologs are shown in cyan; Rec102 homologs and the transducer domain of TOP6BL homologs are shown in blue; Rec104 homologs are shown in green, DNA is shown in yellow and Mg^{2+} ions are in orange. Middle: AlphaFold3 models colored by confidence score. Right: predicted alignment error plot. Despite

bioRxiv preprint doi: https://doi.org/10.64898/2026.01.16.699991; this version posted January 17, 2026. The copyright holder for this preprint (which was not certified by peer review) is the author/funder, who has granted bioRxiv a license to display the preprint in perpetuity. It is made available under aCC-BY 4.0 International license.

extensive variability in the sequence and structure of Rec102 and Top6BL homologs, all the models show Ski8-Rec102 or Ski8-Top6BL interactions, consistent with our interpretation that this interaction is an important pre-requisite for DNA cleavage in fungi. The confidence in the structure and relative position of Ski8-Rec102 and Ski8-Top6BL interactions is indicated by orange squares on the predicted alignment error plots.

bioRxiv preprint doi: https://doi.org/10.64898/2026.01.16.699991; this version posted January 17, 2026. The copyright holder for this preprint (which was not certified by peer review) is the author/funder, who has granted bioRxiv a license to display the preprint in perpetuity. It is made available under aCC-BY 4.0 International license.

Supplementary Table 1: Input sequences and quality scores of AlphaFold models.

Rec104	Substrate	DNA sequence	ipTM	pTM
Full length	Optimal	CGCACACACATCACACACCGCGGTGTGTGATGTGTGTGCG	0.77	0.80
Full length	Consensus	CTAGTAT**C**GAGCTGG**AAGCATGCTT**CTAGATA**G**CGTTAGG	0.51	0.56
Residues 9-58	Optimal	CGCACACACATCACACACCGCGGTGTGTGATGTGTGTGCG	0.85	0.86
Residues 9-58	Consensus	CTAGTAT**C**GAGCTGG**AAGCATGCTT**CTAGATA**G**CGTTAGG	0.49	0.55

The underlined sequence corresponds to the Spo11 cleavage consensus sequence defined by Prieler *et al.*, 2021. This sequence does not yield optimal scores in AlphaFold models. Another sequence that yields higher scores is shown. However, beyond improving model quality, this optimal sequence is unlikely to be biologically meaningful.

Protein	Sequence
Spo11	MALEGLRKKYKTRQELVKALTPKRRSIHLNSNGHSNGTPCSNADVLAHIKHFLSLAANSLEQHQQPISIVFQNKKKKG<br>DTNSPDIHTTLDFPLNGPHLSTHQFKLKRCAILLNLLKVVMEKLPLGKNTTVRDIFYSNVELFQRQANVVQWLDVIRF<br>NFKLSPRKSLNIIPAQKGLVYSPFPIDIYDNILTCENEPKMQKQTIFSGKPCLIPFFQDDAVIKLGTTSMCNIVIVEKEAVF<br>TKLVNNYHKLSTNTMLITGKGFPDFLTRLFLKKLEQYCSNLISDCSIFTDADPYGISIALNYTHSNERNAYICTMANYK<br>GIRITQVLAQNNEVHNKSIQLLSLNQRDYSLAKNLIASLTANSWDIATSPLKNVVIECQREIFFQKKAEMNEIDAGIFKY<br>K
Ski8	MSKVFIATANAGKAHDADIFSVSACNSFTVSCSGDGYLKVWDNKLLDNENPKDKSYSHFVHKSGLHHVDVLQTIER<br>DAFELCLVATTSFSGDLLFYRITREDETKKVIFEKLDLLDSDMKKHSFWALKWGASNDRLLSHRLVATDVKGTTYIW<br>KFHPFADESNSLTLNWSPTLELQGTVESPMTPSQFATSVDISERGLIATGFNNGTVQISELSTLRPLYNFESQHSMINNS<br>NSIRSVKFSPQGSLLAIAHDSNSFGCITLYETEFGERIGSLSVPTHSSQASLGEFAHSSWVMSLSFNDSGETLCSAGWDG<br>KLRFWDVKTKERITTLNMHCDDIEIEEDILAVDEHGDSLAEPGVFDVKFLKKGWRSGMGADLNESLCCVCLDRSIRW<br>FREAGGK
Rec102	MARDITFLTVFLESCGAVNNDEAGKLLSAWTSTVRIEGPEPTDSNSLYIPLLPPGMLKIKLNFKMNDRLVTEEQELFTK<br>LREIVGSSIRFWEEQLFYQVQDVSTIENHVILSLKCTILTDAQISTFISKPRELHTHAKGYPEIYYLSELSTTVNFFSKEGN<br>YVEISHVIPHFNEYFSSLIVSQLEFEYPMVFSMISRLRLKWQQSSLAPISYALTSNSVLLPIMLNMIAQDKSSTTAYQILC<br>RRRGPPIQNFQIFSIPAVTYNK
Rec104	MSIEEEDT**NKITCTQDFLHQYFVTERVSIQFGLNNKTVKRINKDEFDKAVNCIMSWTN**YPKPGLKRTASTYLLSNS<br>FKKSATVSLPFILDDPVCMPKRVESNNNDTCLLYSDTLYDDPLIQRNDQAGDEIEDEFSFTLLRSEVNEIRPISSSSTAQI<br>LQSDYSALMYERQASNGSIFQFSSP

The bold sequence corresponds to Rec104 residues 9-58 that are well-resolved in the cryo-EM structure of the Spo11 core complex (Yu *et al.*, 2024). All models also included four atoms of Mg^{2+}.

bioRxiv preprint doi: https://doi.org/10.64898/2026.01.16.699991; this version posted January 17, 2026. The copyright holder for this preprint (which was not certified by peer review) is the author/funder, who has granted bioRxiv a license to display the preprint in perpetuity. It is made available under aCC-BY 4.0 International license.

Supplementary Table 2: List of plasmids.

Plasmid	Description	Reference
pCCB587	Ski8 in pFastBac1	(Claeys Bouuaert et al. 2021b)
pCCB588	Rec102 in pFastBac1	(Claeys Bouuaert et al. 2021b)
pCCB589	Rec104 in pFastBac1	(Claeys Bouuaert et al. 2021b)
pCCB592	Spo11-HisFlag in pFastBac1	(Claeys Bouuaert et al. 2021b)
pCCB615	Ski8-HisFlag in pFastBac1	(Claeys Bouuaert et al. 2021b)
pCCB617	MBP-Rec102 in pFastBac1	(Claeys Bouuaert et al. 2021b)
pCCB758	REC102::NatMX6 cassette in Topo Blunt II vector	This study
pCCB759	REC104::NatMX6 in Topo blunt vector	This study
pCCB767	Spo11-HisFlag:HphMX4 cassette in pRS314	This study
pCCB771	Spo11-T239A/K240A in pCCB767	This study
pCCB772	Spo11-K126A/N127A in pCCB767	This study
pHAB001	MBP-Tev-Rec102 in pFastBac1	This study
pHAB008	Rec104-H19D in pSK293 (Y2H vector)	This study
pHAB009	Rec104-K39E/R40E in pSK293 (Y2H vector)	This study
pHAB010	Rec104-K48E in pSK293 (Y2H vector)	This study
pHAB011	Rec104-K65E/R66E in pSK293 (Y2H vector)	This study
pHAB012	Rec104-K78E/K79E in pSK293 (Y2H vector)	This study
pHAB013	Rec104-K97E in pSK293 (Y2H vector)	This study
pHAB014	Rec104-R122E in pSK293 (Y2H vector)	This study
pHAB015	Rec104-R147E in pSK293 (Y2H vector)	This study
pHAB016	Rec102-K79E/R81E in pSK282 (Y2H vector)	This study
pHAB017	Rec102-K79E in pSK282 (Y2H vector)	This study
pHAB018	Rec102-R81E in pSK282 (Y2H vector)	This study
pHAB019	Rec102-R89E in pSK282 (Y2H vector)	This study
pHAB020	Rec102-K129E in pSK282 (Y2H vector)	This study
pHAB021	Rec102-H134A in pSK282 (Y2H vector)	This study
pHAB022	Rec102-H136A in pSK282 (Y2H vector)	This study
pHAB023	Rec102-K138E in pSK282 (Y2H vector)	This study
pHAB024	Rec102-K158E in pSK282 (Y2H vector)	This study
pHAB025	Rec102-H171D in pSK282 (Y2H vector)	This study
pHAB026	Rec102-R197E in pSK282 (Y2H vector)	This study
pHAB027	Rec102-R199E/K201E in pSK282 (Y2H vector)	This study
pHAB028	Rec102-K232E in pSK282 (Y2H vector)	This study
pHAB029	Rec102-R243E/R244E/R245E in pSK282 (Y2H vector)	This study
pHAB030	Rec102-R68E in pSK282 (Y2H vector)	This study
pHAB031	Rec102-K64E in pSK282 (Y2H vector)	This study
pHAB032	Rec102-K60E in pSK282 (Y2H vector)	This study
pHAB033	Rec102-K58E in pSK282 (Y2H vector)	This study
pHAB054	MBP-tev-mVenus-Rec102 in pFastBac1	This study
pHAB062	Rec102-K129E in pCCB758	This study
pHAB066	Rec102-R243E/R244E/R245E in pCCB758	This study
pHAB086	Rec102-R245E in pCCB758	This study
pHAB087	MBP-tev-Rec102-R245E in pFastBac1	This study
pHAB092	Spo11-S136A/V138A/E139A in pCCB767	This study
pHAB093	Spo11-F203A in pCCB767	This study
pHAB094	Spo11-F203A/K206A/P207A in pCCB767	This study
pHAB095	Spo11-T239A/N243A in pCCB767	This study
pHAB103	MBP-tev-Rec102-R243E/R244E/R245E in pFastBac1	This study
pHAB119	Spo11-A236V-HisFlag in pFastBac1	This study
pHAB124	Spo11-S136A/V138A/E139A-HisFlag in pFastBac1	This study
pHAB125	Spo11-F203A-HisFlag in pFastBac1	This study
pHAB156	MBP-tev-Rec102-K129E in pFastBac1	This study
pHAB160	Spo11-F203A/T239A/N243A-HisFlag in pFastBac1	This study

bioRxiv preprint doi: https://doi.org/10.64898/2026.01.16.699991; this version posted January 17, 2026. The copyright holder for this preprint (which was not certified by peer review) is the author/funder, who has granted bioRxiv a license to display the preprint in perpetuity. It is made available under aCC-BY 4.0 International license.

pHAB161	Spo11-F203A/T239A/N243A in pCCB767	This study
pHAB162	Spo11-S136A/V138A/E139A/F203A in pCCB767	This study
pHAB164	Spo11-F203A/K206A in pCCB767	This study
pHAB170	Spo11-K240A in pCCB767	This study
pHAB171	Spo11-H246A/K247A in pCCB767	This study
pHAB177	Spo11-H246A/K247A-HisFlag in pFastBac1	This study
pHAB202	Rec102-R243E in pCCB758	This study
pHAB203	Rec102-R244E in pCCB758	This study
pRS314	Yeast shuttle vector (*CEN6 ARS4 TRP1*)	(Sikorski and Hieter 1989)
pSK272	LexA empty Y2H vector	(Arora et al. 2004)
pSK276	Gal4AD empty Y2H vector	(Arora et al. 2004)
pSK282	LexA-Rec102 Y2H vector (pCA1-Rec102)	(Arora et al. 2004)
pSK293	Rec104-LexA Y2H vector (pCA11-Rec104)	(Maleki et al. 2007)
pSK302	Gal4AD-Rec102 Y2H vector (pACT2-Rec102)	(Maleki et al. 2007)
pSK310	Rec104-Gal4AD Y2H vector (pATC2-2-Rec104)	(Arora et al. 2004)
pSK806	SPO11-HisFlag::HphMX6 cassette in pUC19	(Claeys Bouuaert et al. 2021b)

bioRxiv preprint doi: https://doi.org/10.64898/2026.01.16.699991; this version posted January 17, 2026. The copyright holder for this preprint (which was not certified by peer review) is the author/funder, who has granted bioRxiv a license to display the preprint in perpetuity. It is made available under aCC-BY 4.0 International license.

Supplementary Table 3: List of oligonucleotides.

Primer	Sequence
ab001	CAATCCAATGGAAGGACCATGGCAAGAGATATC
ab002	GAAGTACAGGTTTTCCTCGATCCCGAGGTTGTTGTT
ab017	GTACGCAAGACTTTCTTGACCAATACTTTGTAAC
ab018	GTTACAAAGTATTGGTCAAGAAAGTCTTGCGTAC
ab019	GTTAAATAACAAGACCGTAGAAGAGATAAATAAAGATGAATTTG
ab020	CAAATTCATCTTTATTTATCTCTTCTACGGTCTTGTTATTTAAC
ab021	GATAAATAAAGATGAATTTGATGAGGCAGTAAATTGTATC
ab022	GATACAATTTACTGCCTCATCAAATTCATCTTTATTTATC
ab023	GGTACGTTGAAGCTGTCTCTTCTAACCCAGGCTTAGG
ab024	CCTAAGCCTGGGTTAGAAGAGACAGCTTCAACGTACC
ab025	CTTAAGCAATTCCTTTGAGGAATCTGCAACAGTATC
ab026	GATACTGTTGCAGATTCCTCAAAGGAATTGCTTAAG
ab027	CCCAGTATGCATGCCGGAGGAGGTGGAAAGCAATAATAATG
ab028	CATTATTATTGCTTTCCACCTCCTCCGGCATGCATACTGGG
ab029	CGATCCTTTAATACAGGAGAATGATCAAGCAGGAG
ab030	CTCCTGCTTGATCATTCTCCTGTATTAAAGGATCG
ab031	GTGAAGTAAATGAAATCGAACCCATATCTTCGTCC
ab032	GGACGAAGATATGGGTTCGATTTCATTTACTTCAC
ab033	GCAAGAGTTGTTTACAGAATTGGAAGAGATTGTAGGTTCAAG
ab034	CTTGAACCTACAATCTCTTCCAATTCTGTAAACAACTCTTGC
ab035	GCAAGAGTTGTTTACAGAATTGCGCGAGATTGTAG
ab036	CTACAATCTCGCGCAATTCTGTAAACAACTCTTGC
ab037	GTTGTTTACAAAATTGGAAGAGATTGTAGGTTCAAG
ab038	CTTGAACCTACAATCTCTTCCAATTTTGTAAACAAC
ab039	GATTGTAGGTTCAAGTATTGAATTTTGGGAGGAACAAC
ab040	GTTGTTCCTCCCAAAATTCAATACTTGAACCTACAATC
ab041	GATAAGTACGTTCATAAGCGAACCCAGAGAGCTTCATAC
ab042	GTATGAAGCTCTCTGGGTTCGCTTATGAACGTACTTATC
ab043	CAAACCCAGAGAGCTTGCTACGCATGCCAAAGGATATC
ab044	GATATCCTTTGGCATGCGTAGCAAGCTCTCTGGGTTTG
ab045	CAGAGAGCTTCATACGGCTGCCAAAGGATATCCTG
ab046	CAGGATATCCTTTGGCAGCCGTATGAAGCTCTCTG
ab047	GCTTCATACGCATGCCGAAGGATATCCTGAAATC
ab048	GATTTCAGGATATCCTTCGGCATGCGTATGAAGC
ab049	CTGTCAATTTTTTTTCTGAAGAGGGAAACTATGTC
ab050	GACATAGTTTCCCTCTTCAGAAAAAAAATTGACAG
ab051	GAAATAAGCCACGTTATTCCTGATTTTAATGAGTATTTTTC
ab052	GAAAAATACTCATTAAAATCAGGAATAACGTGGCTTATTTC
ab053	CTTCTCCATGATTTCAGAGCTCCGATTGAAGTGG
ab054	CCACTTCAATCGGAGCTCTGAAATCATGGAGAAG
ab055	CATGATTTCAAGGCTCGAATTGGAGTGGCAACAAAGTTC
ab056	GAACTTTGTTGCCACTCCAATTCGAGCCTTGAAATCATG
ab057	CATGATTGCCCAAGACGAATCTTCAACAACCGC
ab058	GCGGTTGTTGAAGATTCGTCTTGGGCAATCATG
ab059	GCGTATCAAATTCTGTGTGAAGAAGAAGGTCCTCCAATTCAGAATTTTC
ab060	GAAAATTCTGAATTGGAGGACCTTCTTCTTCACACAGAATTTGATACGC
ab061	CTGAATTTTAAAATGAACGATGAATTAGTTACGGAAGAGCAAG
ab062	CTTGCTCTTCCGTAACTAATTCATCGTTCATTTTAAAATTCAG
ab063	CCTGGAATGTTGAAAATTAAACTGAATTTTGAAATGAACGATCGATTAG
ab064	CTAATCGATCGTTCATTTCAAAATTCAGTTTAATTTTCAACATTCCAGG
ab065	CCACCTGGAATGTTGAAAATTGAACTGAATTTTAAAATG
ab066	CATTTTAAAATTCAGTTCAATTTTCAACATTCCAGGTGG
ab067	GCTACCACCTGGAATGTTGGAAATTAAACTGAATTTTAAAATGAACG

bioRxiv preprint doi: https://doi.org/10.64898/2026.01.16.699991; this version posted January 17, 2026. The copyright holder for this preprint (which was not certified by peer review) is the author/funder, who has granted bioRxiv a license to display the preprint in perpetuity. It is made available under aCC-BY 4.0 International license.

ab068	CGTTCATTTTAAAATTCAGTTTAATTTCCAACATTCCAGGTGGTAGC
ab133	CCTGTACTTCCAATCGAATTCGGTGAGCAAGGGCGAGGAGCTGTTC
ab134	GTGATATCTCTTGCCATGAGAATTCCCTTGTACAGCTCGTCCATGCCGAGAG
ab135	GACGAGCTGTACAAGGGAATTCTCATGGCAAGAGATATCACATTTTTGACC
ab136	GAACAGCTCCTCGCCCTTGCTCACCGAATTCGATTGGAAGTACAGG
ab143	CCAGGAAAAGTGAAGATTAAACTGAATTTTGAAATGAACGATCGATTAG
ab144	CTAATCGATCGTTCATTTCAAAATTCAGTTTAATCTTCACTTTTCCTGG
ab175	CAACGTGGGCAAAGATGTCCT
ab176	GGACATCTTTGCCCACGTTGT
ab185	CAACGTGGGCAAAGATGTCCTAGCAATGTAATCGTCTATGT
ab186	CATAGACGATTACATTGCTACATGGAGCTGTCTAGAGGATT
ab187	ATCCTCTAGACAGCTCCATGATCACTGGCACTGGTAGAATT
ab188	ATTCTACCAGTGCCAGTGATGGACATCTTTGCCCACGTTGT
ab203	CAACGTGGGCAAAGATGTCCTAGCAATGTAATCGTCTATGT
ab204	CATAGACGATTACATTGCTAGGACATCTTTGCCCACGTTGT
ab216	GGAAAAAGAAGCTGTCTTCGCCAAATTAGTAGCTAATTATCACAAGTTGAG
ab217	CTCAACTTGTGATAATTAGCTACTAATTTGGCGAAGACAGCTTCTTTTTCC
ab218	CAAATTCTGTGTCGAAGAGAAGGTCCTCCAATTCAG
ab219	CTGAATTGGAGGACCTTCTCTTCGACACAGAATTTG
ab228	GCAAAAGCAAACAATTGCCTCTGGTAAGCCCTGTC
ab229	GACAGGGCTTACCAGAGGCAATTGTTTGCTTTTGC
ab230	GCAAAAGCAAACAATTGCCTCTGGTGCGGCCTGTCTAATTCCATTTTTC
ab231	GAAAAATGGAATTAGACAGGCCGCACCAGAGGCAATTGTTTGCTTTTGC
ab234	GAGAGATATCTTCTACGCCAACGCGGCATTGTTTCAAAGACAAG
ab235	CTTGTCTTTGAAACAATGCCGCGTTGGCGTAGAAGATATCTCTC
ab253	GTAATAGTGGAAAAAGAAGTTGTCTTCACCAAATTAG
ab254	CTAATTTGGTGAAGACAACTTCTTTTTCCACTATTAC
ab275	GTAAATAATTATCACGCGTTGAGTACAAATACC
ab276	GGTATTTGTACTCAACGCGTGATAATTATTTAC
ab293	CCAAATTAGTAAATAATTATGCCGCGTTGAGTACAAATACC
ab294	GGTATTTGTACTCAACGCGGCATAATTATTTACTAATTTGG
ab299	GTATCAAATTCTGTGTGAAAGAAGAGGTCCTCC
ab300	GGAGGACCTCTTCTTTCACACAGAATTTGATAC
ab301	CAAATTCTGTGTCGAGAAAGAGGTCCTCCAATTC
ab302	GAATTGGAGGACCTCTTTCTCGACACAGAATTTG
cb95	CTAGTATAGAGCCGGCGCGCCATGTCTAGATAGCGTTAGGTCTGCCGAATAGTACTACTCGGATCCCGAGCGAACCACGC
cb100	GCGTGGTTCGCTCGGGATCCGAGTAGTACTATTCGGCAGACCTAACGCTATCTAGACATGGCGCGCCGGCTCTATACTAG
cb957	TAGCAATGTAATCGTCTATGACGTTAACGTCATAGACGATTACATTGC
cb991	GAAAAATTACCGCTAGGTGCAGCCACTACAGTGAGAGATATC
cb992	GATATCTCTCACTGTAGTGGCTGCACCTAGCGGTAATTTTTC
cb995	CAAACAATTTTCTCTGGTGCGGCCTGTCTAATTCCATTTTTC
cb996	GAAAAATGGAATTAGACAGGCCGCACCAGAGAAAATTGTTTG
cb997	GAAAAAGAAGCTGTCTTCGCCGCATTAGTAAATAATTATC
cb998	GATAATTATTTACTAATGCGGCGAAGACAGCTTCTTTTTC
cb1149	TATCAATGACTTCAACAATC
cb1150	AGCCAGAATTAAAGAGTTTTTC
cb1273	GGACCAAACCGAACCTG
cb1274	GGCATCCAACTTACCTACTG

bioRxiv preprint doi: https://doi.org/10.64898/2026.01.16.699991; this version posted January 17, 2026. The copyright holder for this preprint (which was not certified by peer review) is the author/funder, who has granted bioRxiv a license to display the preprint in perpetuity. It is made available under aCC-BY 4.0 International license.

Supplementary Table 4: List of strains.

Strain	Genotype	Reference
CBY6	*MATa, ho::LYS2, lys2, ura3, leu2::hisG, trp1::hisG*	
CBY7	*MATα, ho::LYS2, lys2, ura3, leu2::hisG, trp1::hisG*	
CBY8	*MATa, ho::LYS2, lys2, ura3, leu2::hisG, trp1::hisG, arg4-bgl*	
CBY11	*MATα, ho::LYS2, lys2, ura3, leu2::hisG, trp1::hisG, arg4-nsp*	
CBY14	*MATa, ho::LYS2, lys2, ura3, spo11Δ::hisG-URA3-hisG*	
CBY15	*MATα, ho::LYS2, lys2, ura3, spo11Δ::hisG-URA3-hisG*	
CBY52	*MATa, ho::LYS2, lys2, leu2::hisG, trp1::hisG, ndt80::kanMX, LexA(op)-LacZ::URA3*	
CBY53	*MATα, ho::LYS2, lys2, leu2::hisG, trp1::hisG, ndt80::kanMX, LexA(op)-LacZ::URA3*	
CBY62	*MATa, ho::LYS2, lys2, ura3, arg4-bgl, SPO11-6His-3FLAG-loxP-HphMX6*	
CBY63	*MATα, ho::LYS2, lys2, ura3, arg4-bgl, SPO11-6His-3FLAG-loxP-HphMX6*	
CBY87	*MATa, ho::LYS2, lys2, ura3, leu2, sae2::LEU2*	
CBY88	*MATα, ho::LYS2, lys2, ura3, leu2, sae2::LEU2*	
CBY312	*MATα, ho::LYS2, lys2, ura3, leu2::hisG, trp1::hisG, arg4-nsp, rec102Δ::URA3*	This study
CBY313	*MATa, ho::LYS2, lys2, ura3, leu2::hisG, trp1::hisG, arg4-bgl, rec102Δ::URA3*	This study
CBY602	*MATa, ho::LYS2, lys2, ura3, leu2::hisG, trp1::hisG, spo11-F203A-HisFlag::HphMX6*	This study
CBY603	*MATα, ho::LYS2, lys2, ura3, leu2::hisG, trp1::hisG, spo11-F203A-HisFlag::HphMX6*	This study
CBY643	*MATa, ho::LYS2, lys2, ura3, leu2::hisG, trp1::hisG, sae2::LEU2, spo11-F203A-HisFlag::HphMX6*	This study
CBY644	*MATα, ho::LYS2, lys2, ura3, leu2::hisG, trp1::hisG, sae2::LEU2, spo11-F203A-HisFlag::HphMX6*	This study
CBY1109	*MATa, ho::LYS2, lys2, ura3, leu2::hisG, trp1::hisG, arg4-bgl, rec104Δ::KanMX*	This study
CBY1110	*MATα, ho::LYS2, lys2, ura3, leu2::hisG, trp1::hisG, arg4-nsp, rec104Δ::KanMX*	This study
CBY1111	*MATa, ho::LYS2, lys2, ura3, leu2::hisG, trp1::hisG, arg4-bgl, spo11Δ::hisG-URA3-hisG*	This study
CBY1112	*MATα, ho::LYS2, lys2, ura3, leu2::hisG, trp1::hisG, arg4-nsp, spo11Δ::hisG-URA3-hisG*	This study
CBY1113	*MATa, ho::LYS2, lys2, ura3, leu2::hisG, trp1::hisG, rec102-K129E::NatMX6*	This study
CBY1114	*MATα, ho::LYS2, lys2, ura3, leu2::hisG, rec102-K129E::NatMX6*	This study
CBY1115	*MATa, ho::LYS2, lys2, ura3, leu2::hisG, sae2::LEU2, trp1::hisG, rec102-K129E::NatMX6*	This study
CBY1116	*MATα, ho::LYS2, lys2, ura3, leu2::hisG, sae2::LEU2, rec102-K129E::NatMX6*	This study
CBY1125	*MATa, ho::LYS2, lys2, ura3, leu2::hisG, rec102-R243E/R244E/R245E::NatMX6*	This study
CBY1126	*MATα, ho::LYS2, lys2, ura3, leu2::hisG, rec102-R243E/R244E/R245E::NatMX6*	This study
CBY1127	*MATa, ho::LYS2, lys2, ura3, leu2::hisG, sae2::LEU2, rec102-R243E/R244E/R245E::NatMX6*	This study
CBY1128	*MATα, ho::LYS2, lys2, ura3, leu2::hisG, sae2::LEU2, rec102-R243E/R244E/R245E::NatMX6*	This study
CBY1129	*MATa, ho::LYS2, lys2, ura3, leu2::hisG, rec102-R245E::NatMX6*	This study
CBY1130	*MATα, ho::LYS2, lys2, ura3, leu2::hisG, trp1::hisG, rec102-R245E::NatMX6*	This study
CBY1131	*MATa, ho::LYS2, lys2, ura3, leu2::hisG, sae2::LEU2, rec102-R245E::NatMX6*	This study
CBY1132	*MATα, ho::LYS2, lys2, ura3, leu2::hisG, trp1::hisG, sae2::LEU2, Recr02-R245E::NatMX6*	This study
CBY1141	*MATa, ho::LYS2, lys2, ura3, leu2::hisG, spo11-S136A/V138A/E139A-HisFlag::HphMX6*	This study
CBY1142	*MATα, ho::LYS2, lys2, ura3, leu2::hisG, spo11-S136A/V138A/E139A-HisFlag::HphMX6*	This study
CBY1143	*MATa, ho::LYS2, lys2, ura3, leu2::hisG, sae2::LEU2, spo11-S136A/V138A/E139A-HisFlag::HphMX6*	This study
CBY1144	*MATα, ho::LYS2, lys2, ura3, leu2::hisG, sae2::LEU2, spo11-S136A/V138A/E139A-HisFlag::HphMX6*	This study
CBY1149	*MATa, ho::LYS2, lys2, ura3, leu2::hisG, spo11-F203A/T239A/N243A-HisFlag::HphMX6*	This study
CBY1150	*MATα, ho::LYS2, lys2, ura3, leu2::hisG, spo11-F203A/T239A/N243A-HisFlag::HphMX6*	This study
CBY1151	*MATa, ho::LYS2, lys2, ura3, leu2::hisG, sae2::LEU2, spo11-F203A/T239A/N243A-HisFlag::HphMX6*	This study
CBY1152	*MATα, ho::LYS2, lys2, ura3, leu2::hisG, sae2::LEU2, spo11-F203A/T239A/N243A-HisFlag::HphMX6*	This study
CBY1185	*MATa, ho::LYS2, lys2, ura3, leu2::hisG, spo11-H246A/K247A-HisFlag::HphMX6*	This study
CBY1186	*MATα, ho::LYS2, lys2, ura3, leu2::hisG, spo11-H246A/K247A-HisFlag::HphMX6*	This study
CBY1187	*MATa, ho::LYS2, lys2, ura3, leu2::hisG, sae2::LEU2, spo11-H246A/K247A-HisFlag::HphMX6*	This study
CBY1188	*MATα, ho::LYS2, lys2, ura3, leu2::hisG, sae2::LEU2, spo11-H246A/K247A-HisFlag::HphMX6*	This study
CBY1213	*MATa, ho::LYS2, lys2, ura3, leu2::hisG, trp1::hisG, rec102-R243E::NatMX6*	This study
CBY1214	*MATα, ho::LYS2, lys2, ura3, leu2::hisG, rec102-R243E::NatMX6*	This study
CBY1215	*MATa, ho::LYS2, lys2, ura3, leu2, trp1::hisG, sae2::LEU2, rec102-R243E::NatMX6*	This study
CBY1216	*MATα, ho::LYS2, lys2, ura3, leu2, trp1::hisG, sae2::LEU2, rec102-R243E::NatMX6*	This study
CBY1217	*MATa, ho::LYS2, lys2, ura3, leu2::hisG, rec102-R244E::NatMX6*	This study
CBY1218	*MATα, ho::LYS2, lys2, ura3, leu2::hisG, rec102-R244E::NatMX6*	This study
CBY1219	*MATa, ho::LYS2, lys2, ura3, leu2, trp1::hisG, sae2::LEU2, rec102-R244E::NatMX6*	This study

bioRxiv preprint doi: https://doi.org/10.64898/2026.01.16.699991; this version posted January 17, 2026. The copyright holder for this preprint (which was not certified by peer review) is the author/funder, who has granted bioRxiv a license to display the preprint in perpetuity. It is made available under aCC-BY 4.0 International license.

CBY1220	*MATα, ho::LYS2, lys2, ura3, leu2, trp1::hisG, sae2::LEU2, rec102-R244E::NatMX6*	This study
CBY1251	*MATa, ho::LYS2, lys2, ura3, leu2::hisG, REC102-WT::NatMX6*	This study
CBY1252	*MATα, ho::LYS2, lys2, ura3, leu2::hisG, REC102-WT::NatMX6*	This study
CBY1253	*MATa, ho::LYS2, lys2, ura3, leu2::hisG, sae2::LEU2, REC102-WT::NatMX6*	This study
CBY1254	*MATα, ho::LYS2, lys2, ura3, leu2::hisG, sae2::LEU2, REC102-WT::NatMX6*	This study
AH6097	*MATa/MATα, ho::LYS2/ho::LYS2, lys2/lys2, ura3/ura3, leu2::hisG/+, trp1::hisG/+, rad50S::URA3/ rad50S::URA3*	This study
AH13528	*MATa/MATα, ho::LYS2/ho::LYS2, lys2/lys2, ura3/ura3, leu2::hisG/+, trp1::hisG/+, rad50S::URA3/ rad50S::URA3, spo11-Y135F-HA3-His6::KanMX/spo11-Y135F-HA3-His6::KanMX4*	This study
AH13529	*MATa/MATα, ho::LYS2/ho::LYS2, lys2/lys2, ura3/ura3, leu2::hisG/leu2 ::hisG, trp1::hisG/+, rad50S::URA3/ rad50S::URA3, spo11-A236V/spo11-A236V*	This study
AH13530	*MATa/MATα, ho::LYS2/ho::LYS2, lys2/lys2, ura3/ura3, leu2::hisG/+, trp1::hisG/+, rad50S::URA3/ rad50S::URA3, spo11-A236V/spo11-A236V*	This study
